# Adsorption and Absorption of Collagen Peptides to Polydimethlysiloxane and Its Influence on Platelet Adhesion Flow Assays

**DOI:** 10.3390/mi11010062

**Published:** 2020-01-05

**Authors:** Matthew G. Sorrells, Keith B. Neeves

**Affiliations:** 1 Chemical and Biological Engineering Department, Colorado School of Mines, Golden, CO 80401, USA; msorrells@mines.edu; 2 Departments of Bioengineering and Pediatrics, University of Colorado Denver|Anschutz Medical Campus, Aurora, CO 80045, USA

**Keywords:** platelets, collagen peptides, cell adhesion, polydimethylsiloxane

## Abstract

Collagen peptides are an alternative to animal derived collagens for platelet function studies under flow. The purpose of this study was to examine the use of collagen peptides in polydimethylsiloxane (PDMS) devices. Three collagen peptides with amino acid sequences and structures that capture von Willebrand factor and bind it with the platelet receptors integrin α_2_β_1_ and glycoprotein VI were patterned on glass, silicon, and PDMS. Each of these surfaces was also functionalized with tridecafluoro-1,1,2,2-tetrahydrooctyltrichlorosilane (FOTS). Surfaces were characterized by their ability to support platelet adhesion, topology by atomic force microscopy, contact angle, and peptides absorption. PDMS readily absorbs collagen peptides, depleting them from solution, thus reducing their adsorption to glass and silicon substrates when used for micropatterning. Treatment of PDMS with FOTS, but not bovine serum albumin or poloxamer 407, inhibits collagen peptide absorption and supports adsorption and platelet adhesion at venous and arterial shear rates. Similarly, FOTS treatment of glass or silicon supports collagen peptide adsorption even in the presence of untreated PDMS. In conclusion, PDMS acts as an absorptive sink for collagen peptides, rendering a non-adhesive surface for platelet adhesion and competing for peptides when used for micropatterning. The absorption of collagen peptides can be overcome by functionalization of PDMS with a fluorinated alkyl silane, thus allowing its use as a material for micropatterning or as a surface for platelet adhesion flow assays.

## 1. Introduction

Platelet adhesion and aggregation are shear stress dependent phenomena. A repertoire of different receptors promote rolling and adhesion to extracellular matrix proteins within the vascular wall and support homotypic and heterotypic cohesion between platelets and other blood cells [1]. A useful research tool to study these interactions are microfluidic flow assays, which we refer to here as the suite of microfluidic devices that have been developed to measure platelet function under physiologic and pathologic hemodynamics [2]. The most common adhesive substrate used in these assays is type I collagen [3], but a promising alternative is collagen related peptides [4], which has advantages both in micropatterning and dissecting the different functional roles of collagen on platelet adhesion and activation.

Type I collagen is found in the vascular wall and plays, at least, three roles in supporting platelet adhesion and activation [5]. First, it binds von Willebrand factor (VWF) in the plasma. VWF is necessary for platelet adhesion at high shear rates (>600 s^−1^) by supporting rolling mediated by the glycoprotein Ib-V-IX complex [6]. Second, it has binding sites for the integrin α_2_β_1_ that supports firm adhesion [7]. Third, it has binding sites for glycoprotein VI (GPVI), which serves as a key signaling receptor for platelet activation [8,9].

Type I collagen derived from animal and human sources has been the primary collagen substrate for platelet adhesion assays in parallel plate flow chambers. These flow chambers were the primary research tool for platelet and leukocyte adhesion from the 1980s to the 1990s [10]. In the 2000s, the parallel plate flow chamber was miniaturized with the growing popularity and accessibility of microfluidics [2]. This transition to smaller channels came with several benefits; lower blood volumes, higher throughput, anatomically inspired geometries, and the ability to micropattern adhesive substrates. Yet as channel sizes became smaller, the ability to homogenously pattern type I collagen emerged as a problem.

Type I collagen fibers are 10–100 µm long and adsorb to substrates in a random pattern [11,12]. In parallel flow chambers with channel widths on the order of 10 mm this was not a problem because a large enough representative area could mask the microscale heterogeneity of the fiber distribution. In microfluidic devices, as the channel width approaches the collagen fiber length, it becomes more difficult to achieve homogeneity. One solution is to mechanically degrade the fibers into smaller fibers, for example, by sonication [13]. Another approach is to deposit the collagen at a high enough concentration to completely coat the surface [14]. However, very high surface concentrations of collagen can provide such a strong platelet activation signal as to mitigate the importance of other platelet autocrine signaling mediated by adenosine diphosphate (ADP) and thromboxane A2. Moreover, large collagen fibers can extend off the surface into the flowing blood, acting as a net for platelets and other blood cells, in an unpredictable manner [4].

Alternatives to native type I collagen fibers include collagen thin films [11], acid soluble collagen [15], type III collagen [16], and collagen peptides [4,17]. Among these alternatives, collagen peptides appear to best mimic the platelet response to type I collagen. Pugh et al. reported that a combination of three peptides—VWF binding peptide (VWF-III), α_2_β_1_ binding peptide (GFOGER), and GPVI binding peptide or collagen related peptide (CRP)—supported platelet adhesion over a physiologic range of shear rates and resulted in platelet aggregates with similar volumes and morphologies as those formed on type I collagen [4]. These peptides, in combination with other adhesive proteins, have been used in microfluidic devices to probe the relative contribution of platelet receptors on adhesion, activation, and aggregation [18].

All prior platelet adhesion studies we are aware of adsorb collagen peptides to glass surfaces that serve as the bottom wall of a flow chamber. However, microfluidic patterning devices and flow chamber walls where adhesive proteins are adsorbed are often made of polydimethylsiloxane (PDMS) [15,19,20,21,22]. In this study, we examine the adsorption and absorption of collagen peptides to devices in which three or four walls of a channel are made of PDMS. Our findings suggest that collagen peptides are absorbed into PDMS, causing negligible patterning of peptides to an assay surface (e.g., glass) when patterning with a PDMS device or onto a PDMS surface. Modification of PDMS with a fluorinated alkyl silane blocks absorption and promotes collagen peptide adsorption, allowing for platelet adhesion and aggregation under flow.

## 2. Materials and Methods

### 2.1. Materials

Bovine serum albumin (BSA) (A9418), 3,3’-dihexyloxacarbocyanine iodide (DiOC6) (D273), glutaraldehyde (340855) heparin salt (H3393), 4-(2-hydroxyethyl)-1-piperazineethanesulfonic acid (HEPES) (H3375), poloxamer 407 (Pluronics® F-127, P2443) were from Sigma–Aldrich (St Louis, MO, USA). 3.2% sodium citrate vacutainers (369714) and 21-gauge Vacutainer^®^ 21 Safety-Lok blood collection sets (367281) were from Becton Dickson (Frankwood Lakes, NJ, USA). 500 µL and 100 uL glass luer lock syringes were from Hamilton (Reno, NV, USA). Tridecafluoro-1,1,2,2-tetrahydrooctyltrichlorosilane (FOTS) (SIT8174.0) was from Gelest (Morrisville, PA, USA). Polydimethylsiloxane and crosslinker were obtained from Krayden (Denver, CO, USA). Collagen related peptides (CRP-XL) [GCO(GPO)10GCOG-amide], 5(6)-carboxyfluorescein (FAM) labeled CRP-XL, GFOGER [GPC(GPP)5GFOGER(GPP)5GPC-amide], and VWF-III [GPCGPP)5GPRGQOGVMGFO(GPP)5GPC-amide] were obtained from Cambcol Laboratories (Cambridgeshire, UK). Glass slides (2” x 3“) were obtained from Fisher Scientific (Lenexa, KS, USA). Pierce Quantitative Fluorometric Peptide Assay (23290) and Texas Red dye (T20175) were from Thermo Fischer (Denver, CO, USA). Phosphate-buffered saline (PBS) was prepared to 137 mM NaCl, 2.7 mM KCl, 10 mM Na_2_HPO_4_, 1.8 mM KH_2_PO_4_, and pH 7.4.

### 2.2. Blood Collection and Preparation

Blood was collected from healthy donors by venipuncture into 4.5 mL, 3.2% sodium citrate (final concentration) vacutainers using a 21-gauge needle. The first vacutainer was discarded. DiOC6 was added to the citrated whole blood to a final concentration of 1 μM and incubated at 37°C for 10 min prior to running flow assays. The study and consent process received institutional review board approval from the Colorado Multiple Institutional Review Board in accordance with the Declaration of Helsinki.

### 2.3. Glass, PDMS and Silicon Substrate Functionalization

Glass slides were immersed in a 50:50 solution of 12 M HCl:methanol for 4 h at room temperature. They were then rinsed with deionized (DI) water, rinsed with isopropyl alcohol, dried with an air brush, and dehydrated in an oven at 60 °C for 4 h. A thin film of PDMS was achieved by pouring a degassed mixture of PDMS base and crosslinker (mixed in 10:1 ratio) into a petri dish. A glass slide was firmly pressed into the petri dish containing PDMS and placed in an oven to cure for 24 h. The slide was removed from the dish and excess PDMS was removed, leaving a thin film of PDMS on one side of the slide. For FOTS functionalization, the PDMS-coated slide was treated with an oxygen plasma for 30 seconds prior to FOTS deposition (Plasma Cleaner, PDC-001, Harrick Plasma, Ithaca, NY, USA). FOTS was vapor deposited onto glass slides, silicon wafers, or slides with PDMS films in a desiccator under vacuum for 12 h.

### 2.4. Microfluidic Patterning Collagen Peptides

A PDMS microfluidic channel (length (l) = 49 mm, width (w) = 100 µm, height (h) = 50 µm) was either left untreated or was blocked with 2% BSA in PBS or 2% Pluronics F-127 for 45 min and then thoroughly rinsed with deionized water, as indicated in the text. To pattern peptides in a strip, the untreated or blocked channel was laid horizontally across glass, PDMS, FOTS-glass, or FOTS-PDMS surfaces. Collagen peptides were mixed together in 10 mM acetic acid to a final concentration of 250 μg/mL each. The microfluidic channel was filled with the peptide solution and incubated for 2 h at room temperature in a petri dish with wet Kim-Wipes® to maintain a humid environment. The channel was then rinsed with 10 mM acetic acid containing 0.1% w/v Texas Red. The Texas Red was used to locate the micropatterned strip of peptides before running the microfluidic assay. The microfluidic device was then gently removed, and the residual liquid was allowed to air dry.

### 2.5. Microspot Patterning Collagen Peptides

To pattern peptides in the absence of a PDMS channel, a 0.5 µL drop of peptides was pipetted in 10 mM acetic acid (250 µg/mL of each peptide) on each of the four surfaces described above and incubated for 2 h at room temperature in a petri dish with wet Kim-Wipes® to maintain a humid environment. The surface was then rinsed with 10 mM acetic acid and dried. 

### 2.6. Whole Blood Microfluidic Assays

A 32 channel (w = 300 µm, h = 50 µm) microfluidic device (Figure S1A) was laid perpendicular to the direction of the microfluidic patterned collagen peptide strip (Figure S2A). In a second set of experiments, a four channel (w = 500 µm, h = 50 µm) microfluidic device (Figure S1B) was laid on top, over the microspot patterned collagen peptides (Figure S2B). Channels were blocked with 2% BSA in PBS for 45 min. Blood was added to reservoirs and perfused through the device at a flow rate to achieve wall shear rates of 300 s^−1^ and 1500 s^−1^ on the bottom wall of the channel for 5 min using a syringe pump (Harvard PhD Ultra, Harvard Apparatus, Holliston, MA, USA). Brightfield and epifluorescent images of platelet accumulation were recorded (40X, NA 0.6, Olympus IX83, Hamamatsu Orca Flash 4.0, Hammamatsu Photonics, Hamamatsu City, Japan). PBS with 10 U/mL heparin was perfused for 2 min to rinse the channels. Platelet aggregates were then fixed by perfusing 2% glutaraldehyde in PBS through the channels for 5 min. The channels were rinsed once more with PBS followed by brightfield and epifluorescent imaging of the final platelet aggregates.

### 2.7. Image Analysis

Custom Python scripts were used to calculate the mean fluorescence intensity (MFI) of each image. Background MFI was defined as the MFI of the first image of the assay where blood had just begun to perfuse through the microfluidic channel, but before significant platelet adhesion. This value was subtracted from each image to correct for background fluorescence. Maximum fluorescence of the assay was used to quantify overall platelet buildup for a given assay.

### 2.8. Atomic Force Microscopy

The morphology of collagen peptides on silicon and PDMS with or without FOTS treatment was measured, as described above, using atomic force microscopy (AFM) (Asylum, MFP 3D, Asylum Research, Santa Barbara, CA, USA). Collagen peptide solutions were incubated on the surfaces using the microspot patterning, as described above, or in 4 mm diameter PDMS wells. A solution containing 250 µg/mL of each peptide (CRP, GFOGER, and VWF-III) in 10 mM acetic was incubated in these wells for 2 h at room temperature in a humid environment. The wells were rinsed in triplicate using DI water, the PDMS device was removed, and the substrate was dried using an air brush. AFM images of the spots were taken using alternating current (AC) Tapping mode in air. The root mean square roughness (RMS) was calculated for each surface using Gwyddion [23].

### 2.9. Contact Angle Measurement

The contact angle of water on different substrates was measured using an in-house interfacial tensiometer constructed by Aman et al. [24]. Briefly, the setup consisted of a cell illuminated with a fiber optic lamp in which a camera and substrate holder were both placed on an optical tubular bench. A 20 μL droplet of DI water was placed on top of each substrate and allowed to settle for 5 seconds. The camera then took pictures of the droplets, and an image processing script was used to measure the contact angle of the water droplets on top of the substrate.

### 2.10. Collagen peptide depletion in PDMS

A PDMS microfluidic device (l = 40 mm, w = 17 mm, h = 50 µm, volume ≈ 35 µL) was laid on top of a PDMS-coated glass slide and filled with a solution of CRP, GFOGER, and VWF-III at a concentration of 250 µg/mL. Peptides were either patterned together (total peptide concentration = 750 µg/mL) on PDMS or FOTS-functionalized PDMS, or they were patterned separately (total peptide concentration = 250 µg/mL) on PDMS. The solution was incubated in the device for 2 h, after which it was recovered from the microfluidic chamber. The concentration of peptides was measured before and after incubation using a peptide-specific fluorometric assay (Quantitative Fluorometric Peptide Assay, Thermo Fischer, Denver, CO, USA).

### 2.11. Visualization of Absorption of Fluorescent Collagen Peptide into PDMS

A PDMS microfluidic device (l = 55 mm, w = 200 µm, h = 50 µm) was laid on top of a glass coverslip. The device and coverslip were either both clean, untreated surfaces or were both treated with FOTS. The microfluidic channel was filled with FAM-labeled CRP-XL at a concentration of 750 µg/mL in 10 mM acetic acid. After 2 h, the channel was rinsed with ten channel volumes of 10 mM acetic acid, removed from the coverslip it was patterned on, and was then moved to a clean coverslip for imaging. Images of the front of the peptides along the wall of the microfluidic channel were taken at z-sections of the channel (Olympus IX-83 equipped with a disc spinning unit, 60X, NA 1.35, Olympus Life Science, Waltham, MA, USA). Maximum intensity projections, line profiles of fluorescence intensity moving into the PDMS bulk, and mean fluorescence values of the projections were all computed using Fiji image processing software [25]. Mean fluorescence intensity was calculated by summing the mean fluorescence intensity of each z-slice for each condition and device, and normalizing by the highest average value for any condition.

## 3. Results

### 3.1. Flow Assays Reveal Sharply Reduced Platelet Adhesion to Collagen Peptides Patterned on or with PDMS

**Figure 1:**
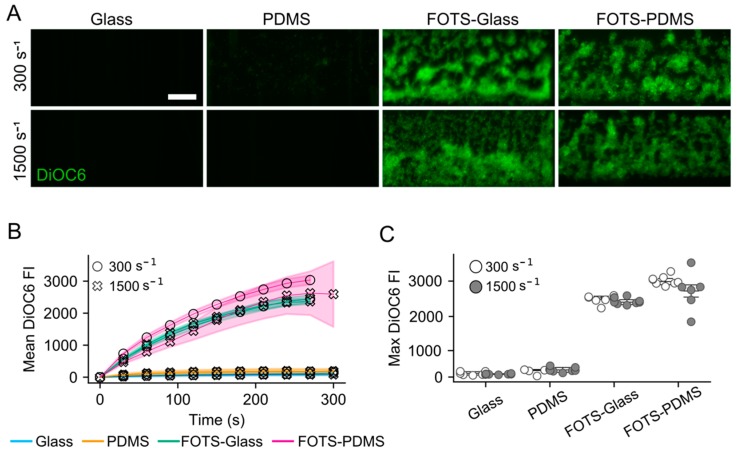
Microfluidic flow assays with polydimethylsiloxane (PDMS) microfluidic patterning. (**A**) Representative images of platelet accumulation on collagen peptides patterned on glass, PDMS, glass functionalized with tridecafluoro-1,1,2,2-tetrahydrooctyltrichlorosilane-glass (FOTS-glass), and PDMS functionalized with FOTS (FOTS-PDMS) after a 5 min perfusion at wall shear rates of 300 s^−1^ and 1500 s^−1^. Scale bar = 50 μm; (**B**) Mean Fluorescence Intensity (MFI) of platelet accumulation over time on the four surfaces. Data points are the average of n = 6, and shaded regions are the standard deviation; (**C**) Maximum mean fluorecence intensity of platelet accumulation over 5 min for the four surfaces.

**Figure 2:**
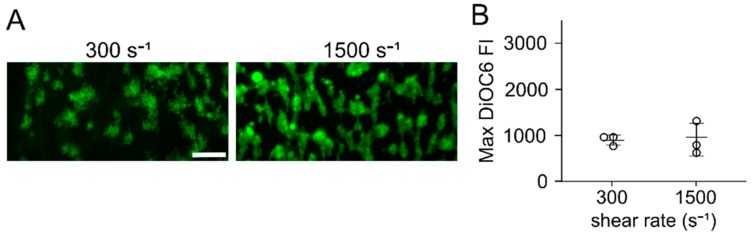
Microfluidic flow assays with microspot patterning. (**A**) Representative images of platelet accumulation on collagen peptides patterned on glass by microspotting after a 5 min perfusion at wall sheare rates of 300 s^−1^ and 1500 s^−1^. Scale bar = 50 µm. (**B**) Maximum fluorescence intensity of assays for n = 3.

Flow assays were run, in which citrated whole blood was perfused over a cocktail of three collagen peptides (CRP, GFOGER, and VWF-III) patterned with an untreated PDMS microfluidic channel or microspotted directly on a surface (no PDMS). When patterning collagen peptides on glass or PDMS using a PDMS microfluidic channel, little to no platelet accumulation at wall shear rates of 300 s^−1^ and 1500 s^−1^ was observed (Figure 1). Similar results were observed when the PDMS channel was first blocked with 2% BSA and 2% Pluronics-F127 prior to patterning. However, when collagen peptides were patterned on FOTS functionalized glass (FOTS-glass) or FOTS functionalized PDMS (FOTS-PDMS) using an untreated PDMS microfluidic channel, platelet adhesion and aggregation at both shear rates was observed. When collagen peptides were microspot patterned on glass, as in de Witt et al. [18], platelet adhesion and aggregation at both shear rates (Figure 2) was observed. These results suggest that the interaction between the collagen peptides and PDMS competes with their adsorption to glass, resulting in a surface that does not support platelet adhesion at the shear rates tested. However, functionalizing the glass or PDMS with FOTS supports platelet adhesion.

### 3.2. Substrate Contact Angles

**Figure 3:**
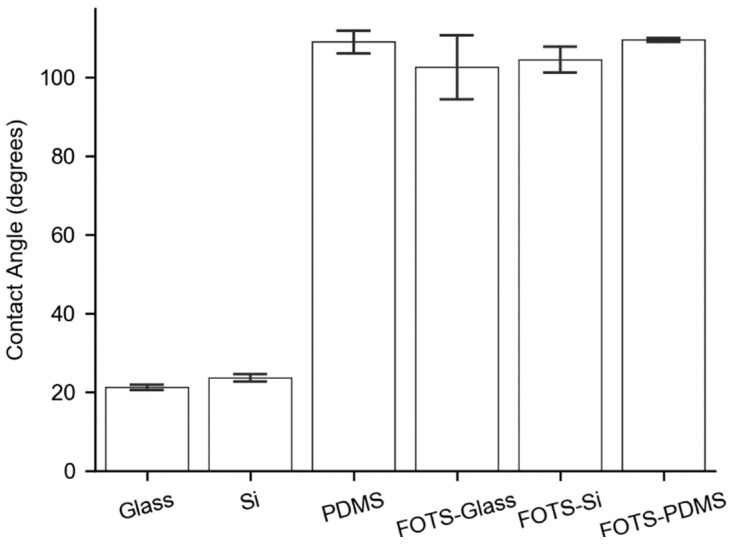
Substrate contact angles. Contact angles at the solid-water-air interface for glass, silicon (Si), native PDMS, and FOTS functionalized glass, silicon, and PDMS.

Because of the differences we observed in platelet accumulation on the different surfaces, we hypothesized that hydrophobic interactions were driving the differences in peptide adsorption on the surfaces. We measured the contact angle of glass, silicon, and PDMS on the clean surface or when functionalized with FOTS. Glass and silicon displayed low contact angles of 22°–23°, while PDMS or all of the FOTS-functionalized surfaces showed contact angles of greater than 100°. We found no significant difference between the contact angle of PDMS and any of the FOTS surfaces (Figure 3). These observations are consistent with other literature values that place the contact angle of PDMS and FOTS at approximately 110° [26,27]. These measurements suggest that hydrophobicity alone is not a sufficient explanation of the differences observed in the flow assays.

### 3.3. Atomic Force Microscopy

**Figure 4:**
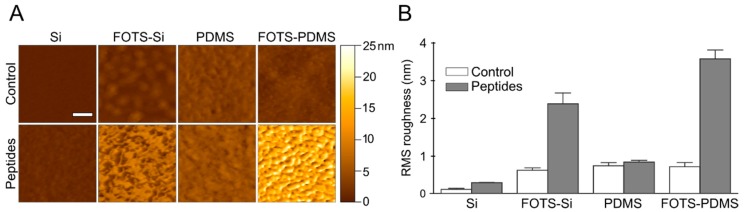
Atomic force microscopy (AFM) of peptide adsorption by microspot patterning. A vehicle control (acetic acid) or collagen peptide solution (250 µg/mL of GPVI binding peptide (CRP), α_2_β_1_ binding peptide (GFOGER), and von Willebrand factor binding peptide (VWF-III) each) was microspot patterned (Figure S2B) on silicon (Si), silicon functionalized with FOTS (FOTS-Si), PDMS, or PDMS functionalized with FOTS (FOTS-PDMS). (**A**) Representative images of each surfaces. Scale bar = 200 nm. (**B**) Average RMS roughness for each surface for n = 3, error bars represent standard deviation.

AFM was used to measure topology of collagen peptides on four surfaces following microspot patterning; silicon, FOTS-silicon, PDMS, and FOTS-PDMS (Figure 4). Silicon was used instead of glass slides for AFM studies because it is smoother and proved better for resolving peptides. The oxide layer on silicon is similar to the hydroxyl terminated surface of glass and displays the same contact angle as glass when untreated or when functionalized with FOTS (Figure 3); however, wettability is only one factor affecting adsorption. Adsorption of peptides on untreated silicon yields a significant change in RMS roughness with features as high as 2 nm. The FOTS-silicon showed a near monolayer of FOTS on the surface with feature sizes in the order of 2 nm (Figure 4B). When patterning peptides on the FOTS-silicon, a three-fold increase in RMS roughness was observed, along with features up to 11 nm high, significantly higher than untreated silicon. Untreated PDMS has a slightly rougher surface than silicon with several nanometer tall features and showed little increase in feature size or roughness following peptide incubation. In contrast, FOTS-PDMS showed features in the order of 20 nm and increased RMS roughness. The observations that collagen peptides adsorb on FOTS-functionalized surfaces is consistent with these surfaces supporting platelet adhesion under flow.

**Figure 5:**
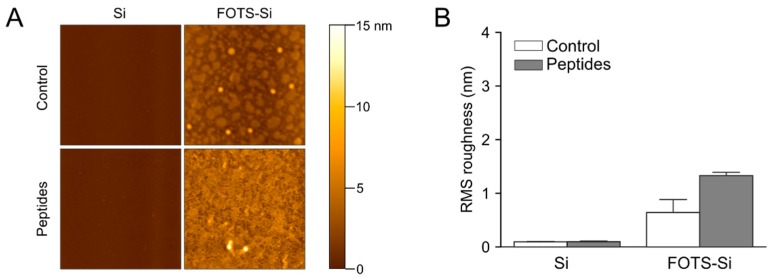
AFM of peptide adsorption by PDMS patterning. A vehicle control (acetic acid) or collagen peptide solution (250 µg/mL of CRP, GFOGER, and VWF-III each) was incubated in a PDMS well on silicon (Si) or silicon functionalized with FOTS (FOTS-Si) (**A**) Representative images of each surface. Scale bar = 3 µm. (**B**) Average RMS roughness for each surface for n = 3, error bars represent standard deviation.

To recreate conditions where a PDMS microfluidic channel was used to pattern peptides for the flow assays, a PDMS well was used to pattern peptides on silicon and silicon-FOTS (Figure 5). On untreated silicon, there was no difference in topology or roughness between incubation with control (10 mM acetic acid) and the collagen peptide solution conditions. This is in contrast to the microspotting technique where no PDMS was present (Figure 4B). On FOTS-silicon, significant peptide adsorption was observed, although the RMS roughness was less compared to microspotting patterning (1.3 nm vs 2.4 nm). These data suggest that PDMS is interacting with collagen peptides in a way that prevents or slows adsorption to silicon, but this can be partially overcome using FOTS functionalization to hasten adsorption.

### 3.4. Depletion of Peptides in Solution

**Figure 6:**
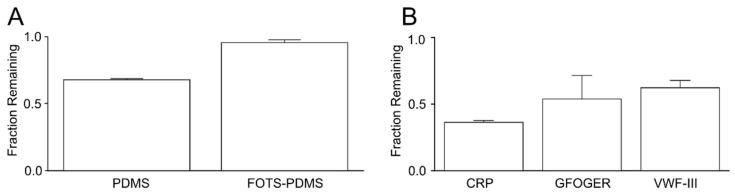
Depletion of collage peptides incubated in PDMS devices. Collagen related peptides were incubated at 250 µg/mL either together (total peptide concentration = 750 µg/mL) or separately (peptide concentration = 250 µg/mL) in a PDMS microfluidic device, and their concentration was measured after 2h. (**A**) Fraction of remaining peptides after incubation on PDMS or FOTS-PDMS. (**B**) Fraction of remaining individual after incubation in PDMS. Error bars show standard deviation of n = 3 measurements.

One possible explanation for the platelet adhesion and AFM data is that PDMS is depleting collagen peptides by absorption. To test this hypothesis, we measured the change in the bulk concentration of three of the collagen peptides after incubation in a microfluidic channel with four PDMS walls for 2 h. We observed a 32% reduction in peptide concentration (Figure 6A). Repeating this experiment with FOTS-PDMS resulted in only a 4% reduction in peptides concentration. We next measured whether there was any preferential depletion between the collagen peptides (Figure 6B). For each peptide, there was a substantial depletion with the CRP having the greatest reduction (64%), followed by GFOGER (46%), and then VWP-BP (35%).

### 3.5. Visualization of Peptide Absorption into PDMS

**Figure 7:**
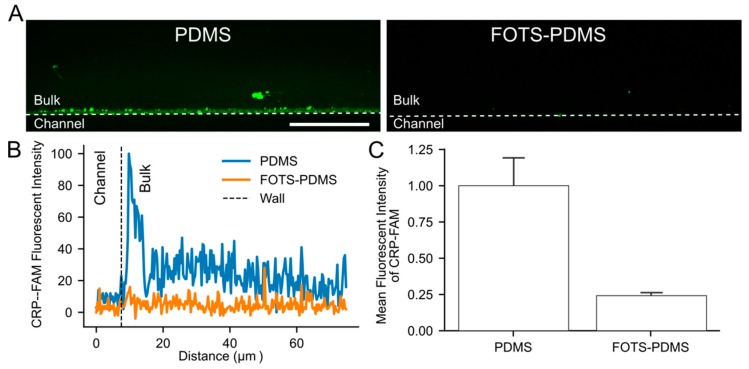
Collagen peptides are absorbed into PDMS but not FOTS-PDMS. (**A**) Representative images of absorption of CRP-FAM (750 µg/mL) into PDMS and FOTS-PDMS following 2 h incubation. The wall of the channel is shown by the dashed white line. Scale bar = 50 µm. (**B**) Line profile of fluorescence intensity showing penetration of CRP-FAM into PDMS and FOTS-PDMS. Dashed black line indicates the location of the channel wall. (**C**) Mean fluorescence intensity of residual CRP-FAM in the bulk of the materials (n = 4).

To visualize the degree of absorption of collagen peptides into PDMS, we incubated a microfluidic channel with a solution of FAM-labeled CRP (CRP-FAM) for 2 h. Penetration of the peptides into the PDMS was observed for untreated PDMS, while no penetration was observed for a FOTS-treated channel (Figure 7A). The CRP-FAM penetrated on the order of 10 µm over 2 h into the bulk of the PDMS, while no penetration was observed in FOTS-PDMS (Figure 7B). The mean fluorescence intensity of CRP-FAM showed a four-fold higher intensity on PDMS compared to FOTS-PDMS (Figure 7C). These results show direct evidence of peptides absorbing into untreated PDMS but not FOTS-PDMS in support of the depletion of peptides in PDMS devices (Figure 6).

## 4. Discussion

In this study, we found that previously developed collagen peptides that support VWF capture and platelet adhesion and aggregation do not adsorb to PDMS in a manner that supports platelet adhesion under flow. Rather, our data suggest that PDMS absorbs collagen peptides. Blocking the microfluidic patterning device with Pluronics F-127 or BSA did not show an increase in platelet adhesion in our flow assays, indicating that blocking the surface with common surfactants used in microfluidics does not effectively disrupt the absorption. However, the absorption of collagen peptides to a PDMS patterning device can be blocked by functionalizing with FOTS. Alternatively, FOTS functionalization of the assay surface (glass or PDMS) provides adsorption that is faster than absorption by the PDMS patterning device. This finding has ramifications, both for using PDMS for micropatterning collagen peptides and for using it as a material for microfluidic flow assays.

The absorption of collagen peptides by PDMS is supported by several lines of evidence. First, platelets do not adhere to PDMS surfaces incubated at a concentration of collagen peptides that support adhesion to glass. Second, there was no observable adsorption of peptides on PDMS at the resolution provided by AFM. Third, incubation of collagen peptide solutions in a monolithic PDMS channel results in significant depletion. Finally, significant penetration of a fluorescently labeled collage peptide was observed in untreated PDMS.

Untreated PDMS absorbs small, hydrophobic molecules, such as some fluorophores and sex hormones [28,29]. Molecules with a logarithmic octanol-water partition coefficient (log P) > 2.62, such as rhodamine 6G and diazepam, are strongly absorbed into PDMS [30]. Similarly, the peptide angiotensin II (~1 kDa) is readily absorbed into native PDMS, which can be attenuated by OEGylation [31]. We are unaware of any prior work showing the absorption into untreated PDMS of triple helix peptides in the molecular weight range (~10 kDa) of those used in this study. It is unknown what properties of peptides might influence their absorption into PDMS, although our data shows that the three peptides used here absorb to different degrees. Whether these observations extends to other peptides, and what the molecular weight cut off is for absorption, requires further research.

The absorption of collagen peptides in PDMS was blocked when the PDMS was functionalized with a fluorinated alkyl silane. This conclusion is based on observations that incubation of collagen peptides on FOTS-PDMS supported platelet adhesion under flow, showed significant topological changes suggesting adsorption, demonstrated negligible depletion of collagen peptide solutions, and showed negligible absorption into the channel wall as visualized with a fluorescently labeled peptide. Whether FOTS blocks absorption by steric hindrance, by promoting hydrophobic interactions, or by some other mechanism requires further investigation. Nevertheless, in practice this simple modification allows PDMS to be used for either micropatterning collagen peptides to glass slides or as a substrate for adsorption itself.

The rate of adsorption of collagen peptides appears to be different for hydrophilic and hydrophobic surfaces. This inference is based on differences observed between untreated and FOTS-functionalized glass and silicon. These findings are supported by molecular dynamics simulations showing that adsorption of a collagen triple helix was more favorable for a hydrophobic surface compared to a hydrophilic surface due to stabilization of the triple helix structure [32]. For untreated glass or silicon, if PDMS was used for patterning collagen peptides, there was little adsorption observed by AFM and negligible amounts of platelet adhesion under flow. For FOTS-glass or FOTS-silicon, significant changes to the surface topology were observe by AFM, and platelet adhesion was supported under flow. These observations suggest that the rate of adsorption to the FOTS-modified surfaces was faster than absorption by PDMS, while the opposite was found for untreated surfaces.

## 5. Conclusions

Microfluidic flow assays are an important tool for studying platelet function. Incorporating repeatable, homogeneous substrates is a desired feature if these assays are to move from research tools to clinical assays. Collagen peptides are an attractive candidate to simulate the response of platelets to subendothelial collagen. Here, we have shown that collagen peptides absorb into PDMS, the most common material used for microfluidic flow assays, yielding a surface that does not support platelet adhesion at venous and arterial shear rates. Importantly, typical blocking reagents like BSA and Pluronics-F127 did not block collagen peptide absorption. However, collagen peptide adsorption can be promoted by treatment of the assay surface with a fluorinated alkyl silane, after which it can be used for micropatterning or for flow assays.

## Supplementary Materials

The following are available online at https://www.mdpi.com/2072-666X/11/1/62/s1, Figure S1: Schematic of microfluidic devices, Figure S2: Procedures for patterning peptides.
