# Supplementary material for: Adsorption and Absorption of Collagen Peptides to Polydimethlysiloxane and Its Influence on Platelet Adhesion Flow Assays
Source: Micromachines (Basel). 2020 Jan 5;11(1):62. doi: 10.3390/mi11010062 (PMC7019490; doi:10.3390/mi11010062)
Supplement: Supplementary file 1 [file micromachines-11-00062-s001.pdf]

Adsorption and Absorption of Collagen Peptides to Polydimethlysiloxane and Its Influence on Platelet Adhesion Flow Assays

Matthew G. Sorrells and Keith B. Neeves

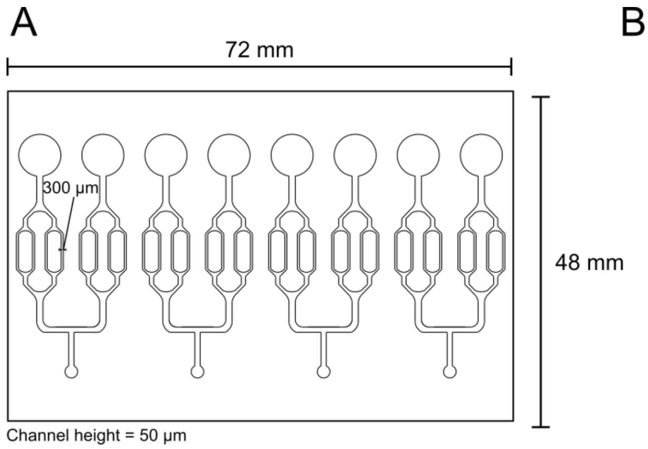

Figure S1. Schematic of microfluidic devices. (**A**) 32 channel microfluidic device used for strip patterning assays. Device channels were 300 µm wide and 50 µm tall. Four Channels were connected to a reservoir, and two reservoirs were connected for a given circuit. Four circuits in total are on the device. (**B**) Four-channel microfluidic device used for microspot patterning. Device consists of four parallel channels with a width of 500 µm and a height of 50 µm.

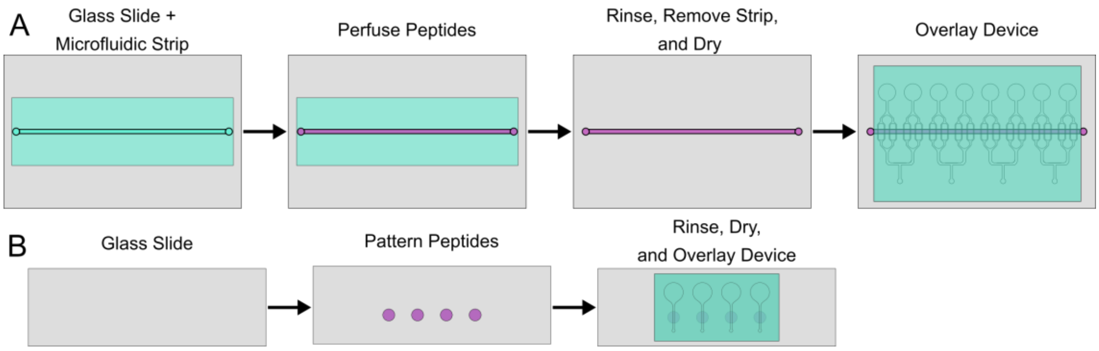

Figure S2: Procedures for patterning peptides. (**A**) Strip patterning procedure. A microfluidic strip is laid across a clean or functionalized glass slide (50 mm x 75 mm), and a solution containing 250 µg/mL of each collagen related peptide in 10 mM acetic acid is perfused through the strip. The peptides are pattern for 2 h at room temperature in a humid environment, after which the strip is rinsed with 10 mm acetic acid, the strip is removed, and the slide is dried. The microfluidic device used for the flow assay is then overlaid such that the strip and the assay region of the device intersect perpendicularly to each other. (**B**) Microspot patterning procedure. A clean or functionalized glass slide is obtained, and four evenly spaced 0.5 uL drops of collagen related peptides at 250 µg/mL in each in 10 mm acetic acid are patterned for 2 h at room temperature in a humid environment. The slide is rinsed with 10 mM acetic acid, dried, and a microfluidic device is overlaid such that each channel intersects with the peptide patch.
